# Protective effect of *Mucuna pruriens* against arsenic-induced liver and kidney dysfunction and neurobehavioral alterations in rats

**DOI:** 10.14202/vetworld.2020.1555-1566

**Published:** 2020-08-12

**Authors:** Preethi Concessao, Laxminarayana Kurady Bairy, Archana Parampalli Raghavendra

**Affiliations:** 1 Department of Physiology, Melaka Manipal Medical College, Manipal Academy of Higher Education, Manipal, Karnataka, India; 2 Department of Pharmacology, RAK College of Medical Sciences, RAK Medical and Health Sciences University, Ras Al Khaimah, United Arab Emirates

**Keywords:** hippocampus, kidney functions, liver functions, memory and learning, *Mucuna pruriens*, sodium arsenite

## Abstract

**Background and Aim:**

Intoxication of arsenic in rats is known to result in neurological effects as well as liver and kidney dysfunction. *Mucuna pruriens* has been identified for its medicinal properties. The aim of the study was to investigate the protective effect of aqueous seed extract of *M. pruriens* on sodium arsenite-induced memory impairment, liver, and kidney functions in rats.

**Materials and Methods:**

The experiment was divided into short-term treatment (45 days) and long-term treatment (90 days), with each group divided into nine sub-groups consisting of six animals each. Sub-groups 1 and 2 served as normal, and N-acetylcysteine (NAC) controls, respectively. Sub-groups 3-9 received sodium arsenite in drinking water (50 mg/L). In addition, sub-group 4 received NAC (210 mg/kg b.wt) orally once daily, sub-groups 5-7 received aqueous seed extract of *M. pruriens* (350 mg/kg b.wt, 530 mg/kg b.wt, and 700 mg/kg b.wt) orally once daily and sub-groups 8 and 9 received a combination of NAC and aqueous seed extract of *M. pruriens (*350 mg/kg b.wt and 530 mg/kg b.wt) orally once daily. Following the treatment, the blood was drawn retro-orbitally to assess the liver (serum alanine transaminase [ALT], serum aspartate transaminase, and serum alkaline phosphatase) and kidney (serum urea and serum creatinine) functions. Learning and memory were assessed by passive avoidance test. Animals were sacrificed by an overdose of ketamine, and their Nissl stained hippocampal sections were analyzed for alterations in neural cell numbers in CA1 and CA3 regions.

**Results:**

In the short-term treatment, groups administered with *M. pruriens* 530 mg/kg b.wt alone and combination of NAC + *M. pruriens* 350 mg/kg b.wt exhibited a significant improvement in memory retention, less severe neurodegeneration, and decrease in serum ALT levels. In long-term treatment, groups administered with *M. pruriens* 700 mg/kg b.wt alone and combination of NAC+*M. pruriens* 350 mg/kg b.wt, respectively, showed better memory retention, decreased neural deficits, and reduced levels of kidney and liver enzymes.

**Conclusion:**

The seed extract of *M. pruriens* showed significant enhancement in memory and learning. The number of surviving neurons in the CA1 and CA3 regions also increased on treatment with *M. pruriens*. Serum ALT, serum urea, and serum creatinine levels showed significant improvement on long-term treatment with *M. pruriens*.

## Introduction

Arsenic is enormously present in underground water throughout the world [1]. The permissible limit for arsenic in drinking water is 10 ppb (WHO) [2]. Abdominal pain, severe diarrhea and vomiting are few symptoms of acute arsenic toxicity [3,4]. Chronic ingestion of arsenic results in its accumulation in vital organs, causing atherosclerosis, hypertension, and peripheral nerve damage [5,6]. Exposure to arsenic affects cognitive function [7-11]. It weakens the cytoskeleton structure, thus damaging the axon [12,13]. Arsenic binds with sulfhydryl groups, producing reactive oxygen species (ROS) resulting in the death of the cell exposed to it [14,15]. It passes through the blood-brain barrier and accrues in the brain [16,17]. Learning and memory decline in humans and animals indicate that the hippocampus is more prone to toxicity caused by arsenic [18,19]. Liver and kidneys are also prone to toxicity since they are essential in metabolic and excretory processes, respectively. It is to be noted that the absorption of arsenic primarily takes place in the small intestine, following which it is found in various organs, including the liver [20-23]. The involvement of the liver is a complication of long-term exposure to arsenic since it tends to get accumulated with repeated exposure [24]. Various studies have shown a connection between continuous exposure to arsenic and liver disease in addition to liver fibrosis, liver cirrhosis, and hepatomegaly [25-30]. Transformation of arsenic from its pentavalent to less soluble and toxic trivalent form occurs in the kidneys. Sites prone to damage are capillaries, glomeruli, and tubules [28-31]. Epidemiological analysis and animal studies have revealed that mild and persistent arsenic exposure causes kidney damage and increase the risk of cancer [32,33]. Further, studies in experimental animals and humans have described that exposure to inorganic arsenic causes damage to the kidney resulting in loss of brush border, tubular necrosis, and renal failure [32,34].

N-Acetylcysteine (NAC), a precursor to glutathione, acts as a biological antioxidant and is used in the treatment of arsenic poisoning [35]. Tissue injury in various organs is prevented due to its ability to scavenge oxygen free radicals [35,36]. The antioxidant property is due to the presence of sulfhydryl group [37,38].

At present, herbal drugs have gained clinical importance since synthetic drugs have shown increased side effects. Antioxidants of plant origin with free-radical scavenging properties could have great importance as therapeutic agents in several diseases caused due to oxidative stress. *Mucuna pruriens* is a leguminous plant which has an anti-lipid peroxidation, anthelmintic, anti-inflammatory, and aphrodisiac and neuroprotective properties [39-42]. In a study done by Sampath *et al.*, [43], it was reported that the neuroprotective property of *M. pruriens* was due to antioxidant activity. A study confirmed that *M. pruriens* could ameliorate kidney damage, which is one of the complications of diabetes mellitus, indicating the nephroprotective property of seed extract [44]. It is also established that the seeds of *M. pruriens* lowered blood urea and creatinine in rats [45]. Further studies have found that hydroethanolic extract of *M. pruriens* demonstrated hepatoprotective activity against anti-tubercular drugs and alcohol models [46]. One of the biggest advantages of plant extracts is that they are less expensive to produce and are affordable to the poor population who need them the most. However, the protective role of *M. pruriens* in arsenic-induced liver and kidney functions, together with neurobehavioral alteration, is not much studied.

Based on the antioxidant and nutritional properties of *M. pruriens*, an attempt was made to investigate the protective effect of aqueous seed extract of *M. pruriens* on sodium arsenite-induced memory impairment as well as liver, and kidney functions in rats.

## Materials and Methods

### Ethical approval

All procedures used in this study were approved by the Animal Ethics Committee of Manipal Academy of Higher Education. (IAEC/KMC/52/2015).

### Study period and location

This study was conducted from September 2018 to March 2019 at Animal House of Manipal Academy of Higher Education and at the Laboratory of Melaka Manipal Medical College, Manipal.

### Chemicals

Sodium arsenite A.R (98.5%) was obtained from Nice Chemicals (P) Ltd, Cochin, India. NAC (Samarth Life Sciences Private Limited, India) was procured from a medical store at Udupi. Thiobarbituric acid, trichloroacetic acid, and DTNB were obtained from Durga Laboratories, Mangalore and the standard kits for liver and kidney function tests were procured from Hitech Biomedicals, Mangalore, India.

### Preparation of plant extract

The identification of *M. pruriens* seeds was carried out by the Faculty of Pharmacognosy (Specimen No: SDM/954/17112301). Seeds of *M. pruriens* were collected locally, cleaned, and ground into a fine powder. 50 g of this powder was macerated in 100 ml distilled water for 1 day at 4°C. Centrifugation of this suspension was carried out at 10000× *g* for 25 min. The supernatant was lyophilized to powder and was stored at −4°C and prepared freshly for use [47]. The yield from the extract was 15.8g.

### Experimental animals

The experimental animals consisted of 108 nos. of male rats (9-12 weeks old) locally bred in the animal house. They were housed in cages with appropriate bedding, standard temperature (22-24°C), light-dark cycle (12 h-12 h), and relative air humidity (40-60%). Rats were acclimated to the laboratory conditions for 7 days before the commencement of the experiment, which was maintained according to the guidelines of CPCSEA, Government of India, for the use of laboratory animals. During the course of the experiment, rats were fed with laboratory feed and water.

### Experimental design

The experiment was divided into short-term treatment (45 days) and long-term treatment (90 days), with each group divided into nine subgroups consisting of six animals each. Subgroups 1 and 2 served as normal and NAC controls, respectively. Subgroups 3-9 received sodium arsenite in drinking water (50 mg/L) [48]. In addition, subgroup 4 received NAC (210 mg/kg b.wt) orally once daily [49], subgroups 5-7 received aqueous seed extract of *M. pruriens* (350 mg/kg b.wt, 530 mg/kg b.wt, and 700 mg/kg b.wt) orally once daily [50] and sub-groups 8 and 9 received a combination of NAC and aqueous seed extract of *M. pruriens* (350 mg/kg b.wt and 530 mg/kg b.wt) orally once daily. The blood was drawn retro-orbitally 24 h after the treatment to assess the liver (serum alanine transaminase [ALT], aspartate transaminase [AST], and alkaline phosphatase [ALP]) and kidney (serum urea and serum creatinine) functions.

These following tests were performed using standard kits.

### Liver function tests

- ALT (modified IFCC procedure)
- AST (Aspartate aminotransferase modified IFCC procedure)
- ALP (modified AMP procedure).

### Kidney function tests

- Creatinine (modified Jaffe’s method)
- Urea (urease/GLDH methodology).

### Behavioral assessment

After the treatment period, memory and learning were evaluated by the passive avoidance method.

### Passive avoidance learning apparatus [51]

It includes three parts: (A) Exploration test, (B) Passive avoidance acquisition, (C) Retention test.

The equipment consisted of a small-sized dark compartment and a large-sized light compartment box separated by a sliding door that could be raised to 10 cm. The stainless steel floor was connected to a shock stimulator that delivered the shock to the grid floor of the dark compartment.

#### (A) Exploration test

The sliding door separating the dark and light compartment was opened, and the rat was let to inspect both the compartments for 180 s. The time in seconds spent by the rat in the small compartment in each trial was recorded. At the end of the trial, the rat was placed in the home cage, where it stayed during an inter-trial interval of 300 s.

#### (B) Passive avoidance acquisition

The rat was placed in the small compartment, and the sliding door between the two compartments was closed. Three strong foot shocks (50Hz, 1.5mA, and 1-s duration) were delivered at a 5-s interval. After the experiment, the rat was placed in the home cage.

#### (C) Retention test

The retention test is carried out 24 h after foot shock. The rat was placed in the middle of the large compartment, facing away from the small compartment. The sliding door between the compartments was opened. The rat explored the small and the large compartments for 3 min, after which it was placed back in the home cage. The trial was repeated thrice with an inter-trial interval of 5 min. In each test, the time duration spent in the small compartment was noted.

A decrease in the time spent in the smaller compartment during retention test is considered as good memory retention performance.

### Cresyl violet staining and neural cell counting

After the behavioral tests, the rats were euthanized and permeated with normal saline, followed by 10% formalin transcardially. The brains were removed and fixed in 10% formalin. 5 μm thick paraffin sections were sliced using a rotary microtome and mounted on slides that were smeared with gelatin. Brain sections from each rat were then stained with 0.1% cresyl violet stain at 60°C for 20 min. Ten sections per rat were selected for quantification. The quantification of surviving neural cells was done in CA1 and CA3 regions of the hippocampus, using a light microscope (Magnus MLX, Microscope). Surviving neural cells with a distinct and clear nucleus were considered for quantification, while darkly stained neural cells with irregular nuclei were not considered for quantification. The quantification was carried after the calibration of a light microscope using an ocular and stage micrometer (Erma, Japan).

### Statistical analysis

The uniform data that were generated were depicted in terms of mean ± standard deviation and were analyzed by one-way ANOVA followed by the *post hoc* Tukey test, and p<0.05 was considered as statistically significant.

## Results

### Passive avoidance test

#### In short-term treatment groups

24 h after foot shock was administered, animals treated with sodium arsenite showed decreased memory retention and spent 74.5% of the total duration in the dark compartment whereas animals in the normal control group spent 37.5% of the total duration in the dark compartment. Group treated with NAC showed results similar to the normal control group. Animals treated with *M. pruriens* 350 mg/kg b.wt, 530 mg/kg b.wt, and 700 mg/kg b.wt spent 69.27%, 55.57%, and 60.5% of the total duration in the dark compartment, respectively, in comparison to sodium arsenite + NAC treated group (60%). Animals treated with a combination of NAC + *M. pruriens* 350 mg/kg b.wt and NAC + *M. pruriens* 530 mg/kg b.wt spent 59.5% and 64.4% of the total time in the dark compartment, respectively. It was observed that there was a significant reduction in the time spent by the animals in the dark compartment in the groups treated with *M. pruriens* 530 mg/kg b.wt alone and a combination of NAC + *M. pruriens* 350 mg/kg b.wt (Table-1).

**Table-1:** Passive avoidance test results - 45 days treatment.

Groups	Retention	

	Time spent in the dark compartment (sec)	No. of crossings
Control	73.67±20.03	1.667±0.51
NAC	75±16.85	1.66±0.81
As control	134.0±7.29*	3.98±0.81*
As+NAC	108.7±8.31	2.16±0.98#
As+MP(350)	124.7±36.04	2.16±1.16#
As+MP(530)	109.7±15.46#	2.33±0.81#
As+MP(700)	104.7±10.73	1.83±0.75#
As+NAC+MP(350)	107.2± 8.29#	2.03±0.63#
As+NAC+ MP(530)	116±9.44	1.16±0.81#

In each group n=6. Values are mean ± SD.

* *P*<0.01: compared to the normal control group; #*P*<0.01 compared to As control group. NAC – N-Acetylcysteine, As- arsenic, MP (350) – *Mucuna pruriens* aqueous seed extract 350 mg/kg body weight, MP (530) - *Mucuna pruriens* aqueous seed extract 530 mg/kg body weight, MP (700) -* Mucuna pruriens* aqueous seed extract 700mg/kg body weight

#### In the long-term treatment groups

24 h after administration of the foot shock, the group treated with sodium arsenite spent 84.4% of the total time duration in the dark compartment showing poor memory retention. Normal controls (42.03%) and NAC (43.15%) treated group showed similar results in the total time duration spent by the animals in the dark compartment. Animals treated with *M. pruriens* 350 mg/kg b.wt, 530 mg/kg b.wt, and 700 mg/kg b.wt spent 61.38%, 62.3%, and 51.1% of the total duration in dark compartment, respectively, when compared to sodium arsenite treated group. Rats treated with *M. pruriens* 700 mg/kg b.wt spent significantly less time in the dark compartment in comparison to *M. pruriens* 350 mg/kg b.wt and 530 mg/kg b.wt groups. It was observed that rats treated with *M. pruriens* 700 mg/kg b.wt alone, and a combination of NAC + *M. pruriens* 350 mg/kg b.wt showed better memory retention (Table-2).

**Table-2:** Passive avoidance test results – 90 days treatment.

Groups	Retention	

	Time spent in small compartment (sec)	No. of crossings
Control	75.67±12.02	1.667±0.51
NAC	77.67±7.98	1.69±0.89
As control	146.0±15.35*	4.12±0.81*
As+NAC	90.5±18.01#	2.06±0.51#
As+MP(350)	110.5±17.08#	2.33±1.21#
As+MP(530)	112±17.17#	2.00±0.63#
As+MP(700)	92±11.51#	1.50±0.54#
As+NAC+MP(350)	89.3±7.35#	2.04±0.21#
As+NAC+MP(530)	98.1±11.08#	2.00±0.16#

In each group n=6. Values are mean ± SD.

* *P*<0.01: compared to the normal control group; #*P*<0.01: compared to As control group. NAC – N-Acetylcysteine, As- arsenic, MP (350) - *Mucuna pruriens* aqueous seed extract 350 mg/kg body weight, MP (530) - *Mucuna pruriens* aqueous seed extract 530 mg/kg body weight, MP (700) -* Mucuna pruriens* aqueous seed extract 700 mg/kg body weight

### Quantitative analysis of hippocampal neurons CA1 and CA3 region

#### Short-term treatment

The CA1 and CA3 regions of the hippocampus showed less surviving neurons in the sodium arsenite treated group when compared to normal control. Animals treated with *M. pruriens* 700 mg/kg b.wt indicated a significant increase in the number of surviving neurons in the CA1 region when compared to the sodium arsenite treated group. It was ascertained that *M. pruriens* 700 mg/kg b.wt alone and a combination of NAC + *M. pruriens* 350 mg/kg b.wt showed an increased number of surviving neurons in the CA1 region in comparison to other treatment groups.

**Figure-1:**
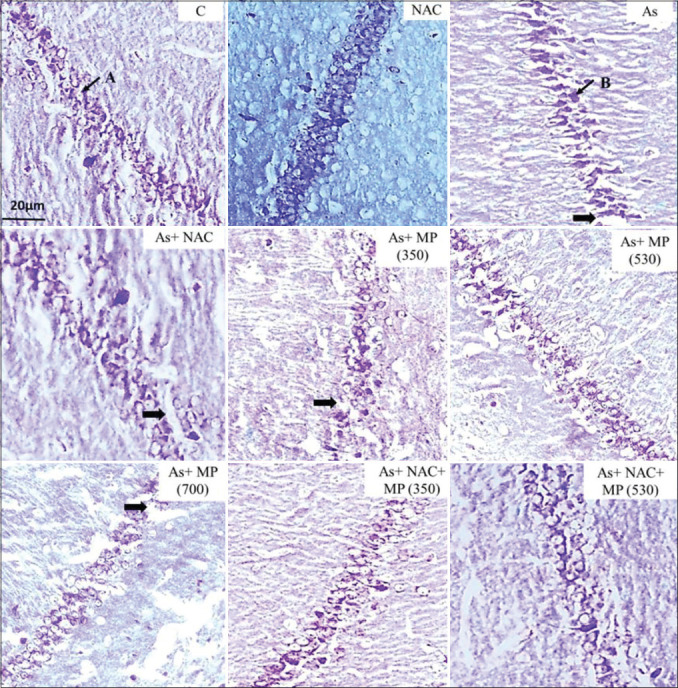
Representative photographs of CV stained sections of CA1 subfield of the hippocampus following 45 days of treatment, viewed under 100×. The layering pattern showed disrupted pyramidal cell layers of (➡) the pyramidal cells in the treatment groups. A: Viable cell. B: Degenerated cell. Scale bar: 20 μm. NAC: N: Acetylcysteine, As: arsenic, MP (350): *Mucuna pruriens* aqueous seed extract 350 mg/kg body weight, MP (530): *M. pruriens* aqueous seed extract 530 mg/kg body weight, MP (700): *M. pruriens* aqueous seed extract 700 mg/kg body weight.

**Figure-2:**
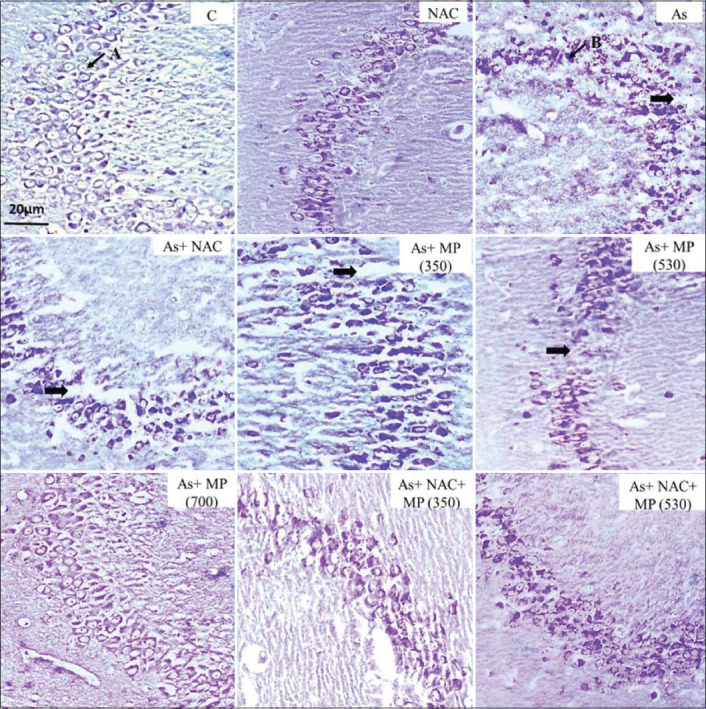
Representative photographs of CV stained sections of CA3 subfield of the hippocampus following 45 days of treatment, viewed under 100×. The layering pattern showed disrupted pyramidal cell layers of (➡) the pyramidal cells in the treatment groups. A: Viable cell. B: Degenerated cell. Scale bar: 20 μm. NAC: N-Acetylcysteine, As: arsenic, MP (350): *Mucuna pruriens* aqueous seed extract 350 mg/kg body weight, MP (530)-*M. pruriens* aqueous seed extract 530 mg/kg body weight, MP (700): *M. pruriens* aqueous seed extract 700 mg/kg body weight.

Animals treated with *M. pruriens* 350 mg/kg b.wt, 530 mg/kg b.wt, and *7*00 mg/kg b.wt indicated an increased surviving neuronal number in the CA3 region when compared to sodium arsenite treated group. The number of surviving neurons in the group treated with *M. pruriens* 700 mg/kg b.wt was similar to the sodium arsenite + NAC treated group. Animals treated with NAC in combination with *M. pruriens* 350 mg/kg b.wt and 530 mg/kg b.wt showed an increased number of surviving neurons in the CA3 region when compared to sodium arsenite +NAC treated group (Table-3, Figures-1 and 2).

**Table-3:** Effect of *Mucuna pruriens* on hippocampal neural cell quantification of sodium arsenite treated rats (45 days). The number of surviving neurons across 100-micron length.

Groups	CA1	CA3
Control	48±7.07	43±10.05
NAC	50±15.3	43±10.22
As control	26±7.33*	23±9.99*
As+NAC	41±8.55	37±9.13
As+MP(350)	38 ±7.34	34±2.57
As+MP(530)	39±8.72	36±4.27
As+MP(700)	43±8.55#	37±2.47
As+NAC +MP(350)	43±6.92#	38±10.22#
As+NAC+ MP(530)	37±5.50	37±2.57

In each group n=6. Values are mean ± SD.

* *P*<0.01: compared to the normal control group; #*P*<0.01: compared to As control group. NAC – N-Acetylcysteine, As- arsenic, MP (350) - *Mucuna pruriens* aqueous seed extract 350 mg/kg body weight, MP (530) - *Mucuna pruriens* aqueous seed extract 530 mg/kg body weight, MP (700) -* Mucuna pruriens* aqueous seed extract 700 mg/kg body weight

#### Long-term treatment

The number of surviving neurons decreased significantly in the hippocampal CA1 and CA3 regions following the administration of sodium arsenite. Normal control and NAC groups showed similar results with respect to the number of surviving neurons in the CA1 and the CA3 regions of the hippocampus. Rats treated with *M. pruriens* 530 mg/kg b.wt and 700 mg/kg b.wt showed a significant increase in the number of surviving neurons in the CA1 region when compared to sodium arsenite treated group. Animals treated with *M. pruriens* 700 mg/kg b.wt alone and combination of NAC + *M. pruriens* 530 mg/kg b.wt exhibited results that were similar to sodium arsenite + NAC treated group. Animals treated with a combination of NAC and *M. pruriens* 530 mg/kg b.wt presented an increase in the surviving neurons when compared to sodium arsenite +NAC group in the CA1 region.

**Figure-3:**
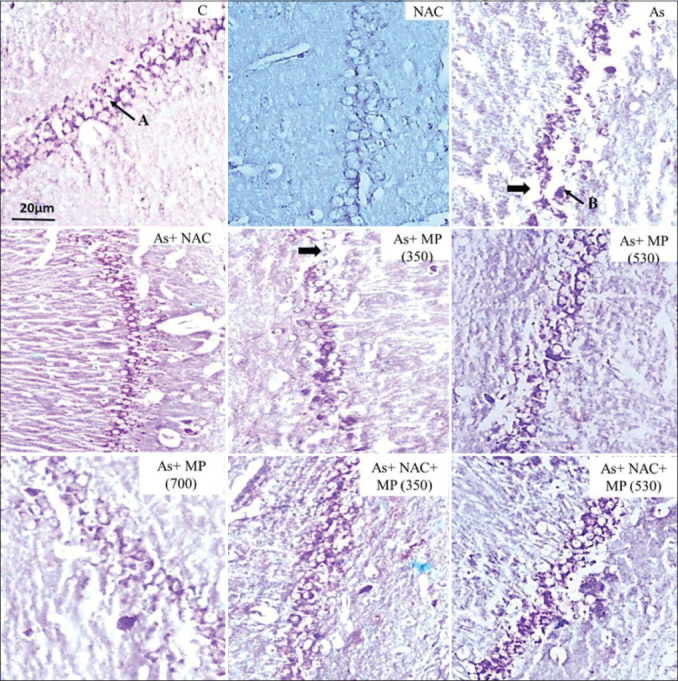
Representative photographs of CV stained sections of CA1 subfield of the hippocampus following 90 days of treatment, viewed under 100×. The layering pattern showed disrupted pyramidal cell layers of (➡) the pyramidal cells in the treatment groups. A: Viable cell. B: Degenerated cell. Scale bar: 20 μm. NAC: N-Acetylcysteine, As: arsenic, MP (350): *Mucuna pruriens* aqueous seed extract 350 mg/kg body weight, MP (530): *M. pruriens* aqueous seed extract 530 mg/kg body weight, MP (700): *M. pruriens* aqueous seed extract 700 mg/kg body weight.

**Figure-4:**
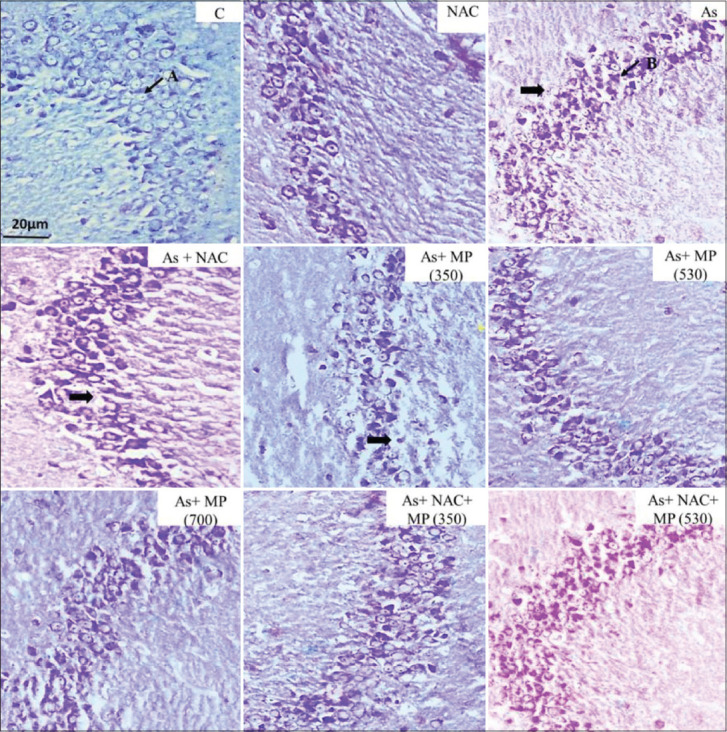
Representative photographs of CV stained sections of CA3 subfield of the hippocampus following 90 days of treatment, viewed under 100×. The layering pattern showed disrupted pyramidal cell layers of (➡) the pyramidal cells in the treatment groups. A: Viable cell. B: Degenerated cell. Scale bar: 20 μm. NAC: N: Acetylcysteine, As: arsenic, MP (350): *Mucuna pruriens* aqueous seed extract 350 mg/kg body weight, MP (530): *M. pruriens* aqueous seed extract 530 mg/kg body weight, MP (700): *M. pruriens* aqueous seed extract 700 mg/kg body weight.

Rats treated with *M. pruriens* 700 mg/kg b.wt alone and a combination of NAC and *M. pruriens* (350 mg/kg b.wt and 530 mg/kg b.wt) showed a significant increase in the number of surviving neurons in the CA3 region when compared to sodium arsenite treated group. Animals treated with *M. pruriens* 350 mg/kg b.wt and 530 mg/kg b.wt showed an increase in the surviving neurons in the CA3 region when compared to sodium arsenite treated group. Rats treated with *M. pruriens* 700 mg/kg b.wt alone and a combination of NAC + *M. pruriens* 530 mg/kg b.wt exhibited an increased number of surviving neurons in the CA3 region in comparison to sodium arsenite + NAC group (Table-4, Figures-3 and 4).

**Table-4:** Effect of *Mucuna pruriens* on hippocampal neural cell quantification of sodium arsenite treated rats (90 days). Number of surviving neurons across the 100-micron length.

Groups	CA1	CA3
Control	51±7.79	41±11.9
NAC	50 ±8.9	41±8.94
As control	20±9.30*	15±8.85*
As+NAC	41±7.07#	33±8.72#
As+MP(350)	36±6.17	29±5.21
As+MP(530)	37±7.07#	30±10.22
As+MP(700)	41±15.07#	32±9.82#
As+NAC+MP(350)	42±4.79#	34±2.29#
As+NAC+MP(530)	41±7.79#	32±4.22#

In each group n=6. Values are mean ± SD.

* *P*<0.01: compared to the normal control group; #*P*<0.01: compared to As control group. NAC – N-Acetylcysteine, As- arsenic, MP (350) - *Mucuna pruriens* aqueous seed extract 350 mg/kg body weight, MP (530) - *Mucuna pruriens* aqueous seed extract 530 mg/kg body weight, MP (700) -* Mucuna pruriens* aqueous seed extract 700 mg/kg body weight

### Effect on liver and kidney functions

#### Short-term treatment

Sodium arsenite administration significantly increased the levels of liver and kidney enzymes in serum when compared to normal controls indicating toxicity. The levels of liver enzymes (AST, ALT, and ALP) showed a decrease in the groups treated with *M. pruriens* 350 mg/kg b.wt, 530 mg/kg b.wt, and 700 mg/kg b.wt in comparison to sodium arsenite treated group, which was not statistically significant. Animals treated with a combination of sodium arsenite + NAC along with *M. pruriens* 350 mg/kg b.wt showed a significant decrease in serum ALT levels when compared to sodium arsenite treated group (Tables-5 and 6).

**Table-5:** Effect of *Mucuna pruriens* (45 days) on liver function tests in the serum of arsenic-treated rats

Groups	AST (U/L)	ALT (U/L)	ALP (U/L)
Control	100.3±9.67	37.78±4.05	107.4±10.22
NAC	105.6±12.61	38.19±3.89	111.0±5.21
As control	140.5±9.13*	49.8±2.30*	159.7±28.86*
As+NAC	110.6±22.28#	40.21±4.31#	139.4±11.99
As+MP(350)	126.6±9.27	45.11±5.43	154.1±8.72
As+MP(530)	124.2±13.62	43.1±5.99	149.6±11.94
As+MP(700)	121±8.85	41.56±5.11	146.5±9.99
As+NAC +MP(350)	119.48±9.32	40.34±3.9#	148±11.9
As+NAC+ MP(530)	117.48±12.52	42.32±3.16	145±10.7

In each group n=6. Values are mean±SD.

* *P*<0.01: compared to the normal control group; #*P*<0.01: compared to As control group. NAC – N-Acetylcysteine, As- arsenic, MP (350) - *Mucuna pruriens* aqueous seed extract 350 mg/kg body weight, MP (530) - *Mucuna pruriens* aqueous seed extract 530 mg/kg body weight, MP (700) -* Mucuna pruriens* aqueous seed extract 700 mg/kg body weight

**Table-6:** Effect of *Mucuna pruriens* (45 days) on kidney function tests in the serum of arsenic-treated rats

Groups	Creatinine (U/L)	Urea (U/L)
Control	0.83±0.11	34.02±3.6
NAC	0.81±0.32	37.19±2.26
As control	1.38±0.12*	45.20±2.40*
As+NAC	1.04±0.26#	41.26±3.33
As+MP(350)	1.17±0.81	42.93±6.88
As+MP(530)	1.08±0.11	43.5±4.80
As+MP(700)	1.10±0.06	42.18±2.68
As+NAC+ MP(350)	1.09±0.05	43.32±2.68
As+NAC+ MP(530)	1.11±0.01	41.26±4.59

In each group n=6. Values are mean±SD.

* *P*<0.01: compared to the normal control group; #*P*<0.01: compared to As control group. NAC – N-Acetylcysteine, As- arsenic, MP (350) - *Mucuna pruriens* aqueous seed extract 350 mg/kg body weight, MP (530) - *Mucuna pruriens* aqueous seed extract 530 mg/kg body weight, MP (700) -* Mucuna pruriens* aqueous seed extract 700 mg/kg body weight.

A decrease in the levels of kidney enzymes (creatinine and urea) in serum was observed in animals treated with *M. pruriens* (350 mg/kg b.wt, 530 mg/kg b.wt, and 700 mg/kg b.wt) along with sodium arsenite, and combination of sodium arsenite +NAC along with *M. pruriens* (350 mg/kg b.wt and 530 mg/kg b.wt) when compared to sodium arsenite group, which was not statistically significant. Animals treated with a combination of sodium arsenite +NAC along with *M. pruriens* 530 mg/kg b.wt had serum urea levels similar to As+ NAC treated group.

#### Long-term treatment

Sodium arsenite treatment significantly increased the levels of liver and kidney enzymes in serum when compared to normal control indicating toxicity. Administration of all doses of *M. pruriens* (350 mg/kg b.wt, 530 mg/kg b.wt, and 700 mg/kg b.wt) along with sodium arsenite led to a significant decline in levels of liver and kidney enzymes in serum when compared to sodium arsenite treated rats. Levels of serum ALT were similar in the group treated with the combination of sodium arsenite + NAC along with *M. pruriens* 530 mg/kg b.wt, and sodium arsenite + NAC group. Animals treated with *M. pruriens* 700 mg/kg b.wt showed serum ALP levels similar to sodium arsenite + NAC group, thus showing a better response.

Animals treated with *M. pruriens* 700 mg/kg b.wt alone and in combination with NAC + *M. pruriens* (350 mg/kg b.wt and 530 mg/kg b.wt) showed a decrease in the levels of serum creatinine when compared to sodium arsenite + NAC group. Treatment with sodium arsenite + *M. pruriens* 700 mg/kg b.wt, and combination of sodium arsenite + NAC along with *M. pruriens* at doses 350 mg/kg b.wt and 530 mg/kg b.wt showed a significant decline in the levels of serum urea. However, the improvement was more significant in rats treated with 700 mg/kg b.wt *M. pruriens* extract (Tables-7 and 8).

**Table-7:** Effect of *Mucuna pruriens* (90 days) on liver function tests in the serum of arsenic-treated rats

Groups	Serum AST (U/L)	Serum ALT (U/L)	Serum ALP (U/L)
Control	100.1±8.55	38.12±4.36	107.1±7.03
NAC	98.89±25.36	37.69±4.60	110.7±15.12
As control	148.5±12.89*	54.7±4.97*	163.4±11.76*
As+NAC	109.5±16.6#	40.26±5.43#	130.9±8.65#
As+MP (350)	125.1±14.18	44.52±2.81#	138.7±19.82
As+MP (530)	123.6±10.16	43.08±2.09#	130.6±16.22#
As+MP (700)	109.8±17.97#	41.17±6.69#	124.1±12.52#
As+NAC+ MP(350)	119.1±12.10	43.35±2.40#	131.6±8.50#
As+NAC+ MP(530)	116.6±8.87#	40.79±6.01#	132.5±7.12#

In each group n=6. Values are mean±SD.

* *P*<0.01: compared to the normal control group; #*P*<0.01: compared to As control group. NAC – N-Acetylcysteine, As- arsenic, MP (350) - *Mucuna pruriens* aqueous extract 350 mg/kg body weight, MP (530) - *Mucuna pruriens* aqueous extract 530 mg/kg body weight, MP (700) - *Mucuna pruriens* aqueous extract 700mg/kg body weight.

**Table-8:** Effect of *Mucuna pruriens* (90 days) on kidney function tests in the serum of arsenic-treated rats

Groups	Serum creatinine (U/L)	Serum urea (U/L)
Control	0.97±0.13	37.2±3.79
NAC	0.99±0.12	37.45±2.55
As control	1.59±0.24*	54.93±3.90*
As+NAC	1.05±0.17#	48.42±2.13#
As+MP(350)	1.22±0.13#	49.61±3.22
As+MP(530)	1.08±0.15#	50.90±2.68
As+MP(700)	0.98±0.12#	42.75±2.69#
As+NAC+MP(350)	1.02±0.26#	46.27±1.50#
As+NAC+MP(530)	1.04±0.24#	44.49±3.37#

Values are mean±SD, (n=6) in each group.

* *P*<0.01: compared to the normal control group; #*P*<0.01: compared to As control group. NAC – N-Acetylcysteine, As- arsenic, MP (350) – *Mucuna pruriens* aqueous extract 350 mg/kg body weight, MP (530) - *Mucuna pruriens* aqueous extract 530 mg/kg body weight, MP (700) - *Mucuna pruriens* aqueous extract 700 mg/kg body weight.

## Discussion

The brain is liable to oxidative deterioration due to an increase in oxygen usage, a high amount of polyunsaturated fatty acids [52], and a lack of antioxidant defense mechanism. Arsenic permeates the blood-brain barrier resulting in damage to the nervous system [53,54]. In this study, rats were exposed to sodium arsenite through drinking water. Passive avoidance test results showed reduced learning and memory in sodium arsenite treated animals along with a decreased number of hippocampal neural cells. Accumulation of arsenic in the hippocampus caused neurodegeneration, thereby bringing about deficits in memory and learning [7,55]. Histopathology showed neurons that were darkly stained with small nuclei. In this study, there was a decrease in the neuronal cell number in the sodium arsenite treated group in comparison to the normal control. In a study carried out by Luo *et al.*, [56], a marked decrease in epinephrine, dopamine, and serotonin was noted in the hippocampus following prolonged exposure to arsenic [57]. Increased neuronal cell apoptosis of the brain has been reported on chronic arsenic exposure [58]. The findings of the subject study showed a decrease in cell number in the hippocampus on arsenic exposure, agreeing with the previously reported work. Arsenic exposure increases the production of free radicals, which damages the lipids, proteins, and DNA [3], leading to a reduction in the cognitive process and also blocks the sulfhydryl groups of enzymes and proteins [59]. The neurodegeneration caused by arsenic is due to synaptic injury, free radical production, and lipid peroxidation [60]. In a study carried out by Tavarekere *et al.*, [61], it was found that learning and memory deficits that were observed were due to a decrease in the acetylcholinesterase activity in the brain following arsenic exposure. The previous studies have demonstrated arsenic-induced neuronal cell death through potential mechanisms like oxidative stress [62-64].

Liver cell integrity is measured by estimating the AST and ALT enzyme levels. Enzyme levels of ALP are a measure of albumin and bile synthesis by the liver. Studies have told that increased measures of AST and ALT in the plasma of rats exposed to arsenic are mainly due to damage to the hepatic cell membrane. This damage increases membrane permeability resulting in leakage of cellular contents [19,65]. Following previous reports, we observed significantly higher levels of AST and ALT in rats exposed to arsenic when compared to healthy controls. Arsenic toxicity leads to inhibition of cellular respiration in mitochondria [66], enhancing the generation of ROS. This further causes an increase in lipid peroxidation and damage to the cells [67].

Studies have reported an association between the rise in arsenic levels in the blood and elevated concentrations of serum urea, creatinine, and damage to the tubules [68,69]. Animal experiments and epidemiologic studies in humans have clearly shown that acute and chronic arsenic exposure can damage the kidneys and increase the risk of cancer [70]. According to Gora *et al.*, [69], there was an exceptional increase in the BUN and creatinine levels following exposure to arsenic in rats. The proximal convoluted tubule cells are sensitive to toxicity induced by arsenic due to their reabsorptive function [20]. Arsenic reacts with compounds containing the sulfhydryl group [59]. It inhibits the GSH reductase and produces excessive ROS in the kidney, which will lead to damage of the respective organ [71]. According to the results in this study, the increased concentrations of serum urea and serum creatinine in 90 days treatment support greater involvement of the renal tubular cells in the excretion of arsenic, making them prone to damage.

Seeds of *M. pruriens* contain bioactive compounds such as flavonoids, alkaloids, tannins, and phenolic compounds [72]. It also contains levodopa, which can cross the blood-brain barrier and restore neurotransmission [39]. It has a positive effect on learning and memory. Polyphenols and quercetin, which are the components of *M. pruriens*, exert their anti-inflammatory and iron-chelating properties in the brain [73]. Besides, in MPTP treated animals, treatment with *M. pruriens* suppressed the inflammatory response due to glial cell activation and dopaminergic neuronal loss [74]. It also exhibits a potent antioxidant, anti-inflammatory, and free-radical scavenging activity. Flavonoids are responsible for the radical-trapping properties and are efficient against the destruction that is mediated by ROS [75]. Thus, it can protect liver tissue from arsenic-induced ROS and prevents inflammation and hepatic cellular death in *M. pruriens* extract-treated group. *M. pruriens* seed extract decreased the lipid peroxidation in the liver and kidney tissue of diabetic rats in a dose-dependent manner [76].

Even though increased arsenic levels in drinking water (above 50 μg/L) have been reported in many countries such as Nepal, Argentina, China, Japan, and Vietnam, the main stirred areas are situated in Bangladesh and West Bengal in India. The arsenic concentrations in groundwater in these areas have been documented up to 3200 μg [77,78]. Since arsenic-rich drinking water is inevitable in these areas, the susceptibility of organ toxicity further leading to damage is also unavoidable. Handling of toxicity is to be done in such a way that the body should not be burdened further. In this context, plant-based treatment may help, as the body can easily handle them without side effects. Further, *M. pruriens* is proved to have nutritional and medicinal values [39]. Therefore, *M. pruriens* can be administered in the diet of people affected by arsenic so that both the nutritional and medicinal needs are taken care of.

## Conclusion

Administration of *M*. *pruriens* with sodium arsenite improves memory retention and prevents neurodegeneration of the CA1 and CA3 regions of the hippocampus in short- and long-term treatment groups. It also improves the liver and kidney functions in long-term treatment groups. It can be concluded that these beneficial effects of *M*. *pruriens* are due to its antioxidant property.

## Authors’ Contributions

PC and LKB designed the study. PC performed the research and drafted the manuscript. PC and LKB analyzed the data. PC, LKB, and APR revised and finalized the manuscript for submission. All authors read and approved the final manuscript.

## Competing interests

The authors declare that they have no competing interests.

## Publisher’s Note

Veterinary World remains neutral with regard to jurisdictional claims in published institutional affiliation.
